# Patenting human genes: Chinese academic articles’ portrayal of gene patents

**DOI:** 10.1186/s12910-018-0271-8

**Published:** 2018-04-24

**Authors:** Li Du

**Affiliations:** 1 Faculty of Law, University of Macau, E32, Avenida da Universidade, SAR Taipa, Macau; 2 Centre for Biomedical Ethics, Fudan University, Shanghai, China

**Keywords:** Human gene patents, Chinese scholars, Patentability

## Abstract

**Background:**

The patenting of human genes has been the subject of debate for decades. While China has gradually come to play an important role in the global genomics-based testing and treatment market, little is known about Chinese scholars’ perspectives on patent protection for human genes.

**Methods:**

A content analysis of academic literature was conducted to identify Chinese scholars’ concerns regarding gene patents, including benefits and risks of patenting human genes, attitudes that researchers hold towards gene patenting, and any legal and policy recommendations offered for the gene patent regime in China.

**Results:**

57.2% of articles were written by law professors, but scholars from health sciences, liberal arts, and ethics also participated in discussions on gene patent issues. While discussions of benefits and risks were relatively balanced in the articles, 63.5% of the articles favored gene patenting in general and, of the articles (*n* = 41) that explored gene patents in the Chinese context, 90.2% supported patent protections for human genes in China. The patentability of human genes was discussed in 33 articles, and 75.8% of these articles reached the conclusion that human genes are patentable.

**Conclusion:**

Chinese scholars view the patent regime as an important legal tool to protect the interests of inventors and inventions as well as the genetic resources of China. As such, many scholars support a gene patent system in China. These attitudes towards gene patents remain unchanged following the court ruling in the *Myriad* case in 2013, but arguments have been raised about the scope of gene patents, in particular that the increasing numbers of gene patents may negatively impact public health in China.

## Background

The rapid progress of genetic diagnostic technology, along with the advent of inexpensive gene sequencing, has increased the availability and affordability of DNA-related tests and genome-based treatment for patients [1]. Following the lead of the United States (US), China has proactively joined the global trend in inventing genetic testing techniques and has become an emerging market for genetic testing services. A recent study estimated that the gene-sequencing market in China will reach nearly $2.5 billion USD by 2021 [2]. The Beijing Gene Institute (BGI), the international center for DNA-sequencing services, is expanding its focus from genomics research and development to pharmaceutical drug discovery and precision-medicine initiatives [3, 4].

Similar to the business model of other pharmaceutical innovations, intellectual property law has played a key role in securing a path forward to the commercialization of genetic diagnostics based on mutated segments of DNA. That being said, the introduction of genetic testing has raised challenges under formal intellectual property laws and general legal rules [5, 6]. A fundamental question that has dominated the controversies over gene patents in the past decade is whether a human gene is considered eligible material for a patent protection [7–9]. The patentability of human genes has been scrutinized in many jurisdictions, including the US, Canada, Australia, and the European Union (EU) [9–11]. In 2013, the Supreme Court of the US denied the patent-eligibility for isolated genetic sequences in *Association for Molecular Pathology v. Myriad Genetics* (*Myriad* Case) [12]. The case has been regarded as a milestone in the practice of gene patenting in the US, and the Court’s ruling captured global attention and triggered significant debate [12, 13]. Similarly, in 2015, Australian judges ruled that an isolated gene was a discovery rather than a patentable invention [14]. In the case of *D’Arcy v. Myriad Genetics*, the High Court of Australia ruled that the BRCA 1 genes associated with the development of breast and ovarian cancers cannot be patented [14, 15]. Although Myriad’s gene patents are still valid in many other countries, commentators anticipate that judges in those jurisdictions might follow the precedence of these suits and disallow the patentability of human genes [11].

In China, the 2000 *Patent Law of the People’s Republic of China* (*Patent Law*) excludes a mere discovery of nature from being granted a patent right [16]. Nonetheless, whether a discovery of naturally occurring human genes is patentable remained uncertain until the *Guidelines for Patent Examination* (*Guidelines*) was issued by the State Intellectual Property Office of the People’s Republic of China in 2010 [17]. Item 9.1.2.2 of the *Guidelines* deals specifically with the issues associated with the patentability of genes in China. This item regulates that a human gene or DNA segment is patentable if: 1) it is isolated or extracted from the natural sequence for the first time, and 2) the use-value of the gene for industry is accurately expressed [17]. Accordingly, isolated genes with an identified practical application are patentable under the existing Chinese patent regime. However, it is unclear whether or not China will follow the US and Australian courts’ ruling and invalidate the granted gene patents. To date, no legal documents have been updated since the 2010 *Guidelines*, and in legal practice, there have not been any reported lawsuits filed for infringement of human gene patents.

A few studies have addressed Chinese patent protection on biotechnology inventions. For example, in 2005, Liu explored the motivations of the Chinese patent system from a historical perspective with a focus on societal issues associated with the implementation of a strict patent protection for biotechnology innovations in China [18]. In a 2014 study, Li and Cai discussed the scope of patent protection for gene technology in China, arguing that the scope should be wider than the existing protection [19]. However, a full picture of Chinese scholars’ argument about the legal and ethical issues concerning gene patents is not clear. For example, little is known about their attitudes towards patenting human genes and their views of the Chinese patent protection regime for genes, and in particular, whether Chinese scholars have begun to change their views towards gene patents in response to the *Myriad* cases started in 2010 and the US and Australian courts’ final decisions striking down gene patents. To fill this gap, I conducted a content analysis of academic research articles to explore Chinese scholars’ key concerns about patenting human genes and highlight trends and the potential future of China’s approach to gene patents. I analyzed 63 Chinese academic articles published from 2000 to 2016 that discussed patent protections for human genes and identified the positions and arguments that researchers held and made regarding the gene patenting.

## Methods

### Search strategy

Using the China Academic Journal Network Publishing Database (CAJD), the most comprehensive database of academic knowledge resources in China with a record of journals published since 1915, I collected Chinese-language journal articles published between 2000 and 2016 that focused on human gene patent issues.

The dataset was compiled by searching the keywords “gene patent” (in Chinese “基因专利”) by subject and not containing words: “animal” (in Chinese “动物”) and “plant” (in Chinese “植物”) in the full text. CAJD covers multiple disciplines including the natural sciences, agriculture, engineering, philosophy, medicine, and humanity and social sciences. This primary retrieval resulted in 91 articles.

### Selection criteria

Given that the CAJD covers more than 8300 academic journals, the written style and format of publications varies significantly [20]. To ensure the quality of the journal articles in the dataset, I adopted the General Catalogue of Chinese Core Journals (GCCCJ, in Chinese: “《中文核心期刊要目总览》”), a journal list produced by the Peking University, as a reference index during the content analysis [21]. In addition, I excluded articles from the dataset that were written in a news report style and had no abstract and keyword component. Moreover, as this study is only concerned with academic publications on human gene patents, articles that were indirectly related to the gene patent discussion such as the patenting of genes of genetically modified foods, or genes of a certain deer species were excluded from the dataset. As a result, the eligibility criteria excluded 28 articles from the dataset, and the final research dataset consisted of 63 academic journal articles.

### Content analysis

A coding framework was developed based on an exploratory thematic analysis of 30% of the dataset and then applied to the entire dataset. The coding framework consisted of the following 16 questions: 1) When was this article published? 2) What is the background of the author(s)? 3) Is the journal indexed by the GCCCJ of the 2000, 2004, 2008, 2011 or 2014 editions? 4) Does the article mention benefits of gene patenting? 5) If yes, what are they? 6) Does the article discuss negative impacts associated with gene patenting? 7) If yes, what are they? 8) Does the article argue that genes are eligible subject for patenting? 9) If yes, what are the reasons? 10) If it argues that genes are not patentable, what are the reasons? 11) Does the article support gene patenting in China? 12) If it supports gene patenting, what are the reasons? 13) If it is against gene patenting, what are the reasons? 14) Does the article suggest measures to improve the Chinese IP system for the patenting of genes? 15) If yes, what are they? 16) What is the attitude of the author(s) towards gene patenting in general?

### Quality assessment

The author coded the entire dataset of articles and invited an independent scholar to code 30% of the dataset (*n* = 18) in order to calculate inter-coder agreement. The author used Cohen’s Kappa to evaluate the agreement. Kappa scores ranged from 0.68 to 0.84 indicating substantial to perfect agreement [22].

## Results

### Article information

**Fig. 1:**
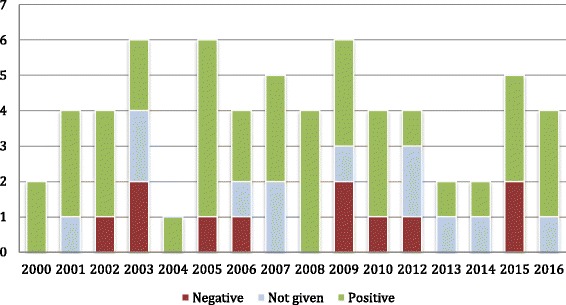
Number of articles published each year and the attitudes toward gene patenting in general

**Fig. 2:**
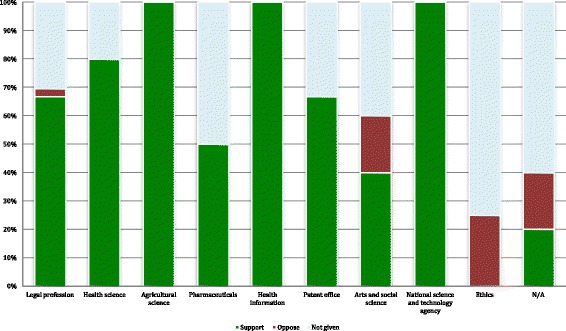
Percentages of attitudes towards a human gene patent protection in China

From 2000 to 2016, the data show that the debate around patenting human genes attracted a comparatively high volume of discussions in 2003, 2005, and 2009 with six articles published each year. No articles in the dataset were produced in 2011, and only one article was published in 2004. There were two articles published in 2000, 2013 and 2014, and for the rest of the years observed, approximately four to five articles were published per year (see Fig. 1). With regard to the authors’ backgrounds, 57.2% of articles (*n* = 36) were written by scholars who identified themselves as part of the legal profession. The authors of the rest of the articles were mainly from three disciplines: liberal arts (7.9%, *n* = 5), health sciences (7.9%, n = 5), and ethics (6.3%, *n* = 4) (see Fig. 2). 36.5% of the articles (*n* = 23) were published in journals indexed by the GCCCJ list.

### Benefits and negative impacts

Benefits associated with the patenting of human genes were mentioned or discussed in 69.8% of articles (*n* = 44). The most frequently cited benefit was that the patent protection of genes can promote inventions and protect inventors (mentioned or discussed in 35 or 55.5% of articles). The next most frequently cited benefit was that the commercialization of gene-based technology will bring huge market profits and the patent protection regime will play a key role in reaping the profits in global markets (mentioned or discussed in 19 or 30.2% of articles). 11 articles (17.5% of articles) mentioned that gene patents can help China become one of the leading players in the gene-related global industry (see Table 1).

**Table 1:** Benefits and negative impacts associated with gene patents

Benefits	Mentioned times
• Promoting inventions	21
• Huge market profits	19
• Protecting inventors	14
• Becoming a dominant role in the gene-related industry	11
• Possessing genetic resources	4
Negative impacts	
• Impeding gene-related research and development	20
• Monopolizing genetic testing services	17
• Impeding access to healthcare	11
• Depriving the rights of providers of human genetic resources	11
• Impacting human being’s autonomy, privacy, dignity, and opposing the social justice	6
• Violating public policy and morality	5
• Robbing the genetic resources of developing countries	4
• Violating the non-discrimination principle in WTO	1
• Impacting international collaboration	1

In terms of negative impacts, 53.4% of articles (*n* = 40) raised concerns about the potential risks of gene patenting. Different types of risks were raised in the articles, e.g., 31.7% of articles (*n* = 20) warned that gene patenting might impede gene-based research and related development. Other frequently cited risks were that gene patenting may lead to the monopolization of genetic testing services (*n* = 17 or 27.0% of articles), impede patients’ access to healthcare (*n* = 11 or 17.5% of articles), and result in the denial of human genetic resource owners’ rights (*n* = 11 or 17.5% of articles) (see Table 1).

### Discussions of patentability of human genes

Controversies over the patentability of human genes, i.e., whether human genes can be considered eligible for patenting were discussed in 33 articles. Of these articles, 75.8% (*n* = 25) reached the conclusion that human genes are patentable. Among these supportive articles, six articles were published after 2010 and 19 articles were published in or before 2010. As previously mentioned, before the 2010 *Guidelines*, it was uncertain whether a discovery of naturally occurring human genes was patentable. Given this context, 16 articles published in or before 2010 had developed the discussions on the patentability of human genes by examining three standards for granting a patent specified in the 2001 *Patent Law*, namely the novelty, inventiveness, and practical applicability. They emphasized the importance of the practicability standard and favored patent protections for genes as they have practical applications. Of the six articles published after 2010, four articles based their supportive arguments on the patentability of human genes on the examinations of the three standards. One 2016 article argued that the Chinese context, i.e., the level of development of genetic technology in China, requires that human genes be patentable.

By contrast, 24.2% of the articles that explored the issue of patent-eligibility for human genes (*n* = 8) argued against the patentability of human genes. Of these articles, two articles were published after the 2010 *Guidelines*, which entitles isolated DNA sequences with clearly stated practical applications patentable. These two articles both argued that gene sequences should not be patentable because they are discoveries of nature rather than inventions. This argument had been used in three other articles published in or before 2010. In total, four articles claimed that genes are owned by human beings and should not be considered eligible for patenting. One 2015 article raised concerns on potential negative influences on public interests due to gene patents and opposed the patentability of human genes.

### Attitudes towards the gene patenting

The review found that 63.5% of articles (*n* = 40) favored gene patenting in general, while 17.5% of articles (*n* = 11) objected to the patenting of human genes. 19.0% of articles (*n* = 12) provided no explicit opinions on gene patents (see Fig. 1). Among the articles published in GCCCJ indexed journals, 11 articles were supportive of gene patents, while eight articles were against and four provided a neutral perspective. For the articles that supported gene patenting, 67.5% (*n* = 27) were authored by members of the legal profession. It is worth noting that articles written by government patent offices (*n* = 3) *all* showed a positive attitude towards gene patenting in general. On the contrary, of the four articles authored by ethics scholars, three articles objected to the patenting of human genes, and one article did not express an explicit opinion. Only two articles contributed by the legal profession argued against gene patents.

When discussing issues related to the patenting of human genes in the Chinese context, 41 articles expressed their opinions on whether China should recognize gene patents. Among these articles, overwhelmingly, 90.2% of the articles (*n* = 37) took a supportive position for human gene patenting in China (see Fig. 2). The positive claims were formed based on various considerations. In addition to the claim that patent systems can promote inventions and protect inventors (discussed or mentioned in 32 articles), researchers also provided other arguments in support of gene patents, e.g., gene patents can contribute to protecting developing countries’ genetic resources (discussed or mentioned in 12 articles), and gene patents can help increase China’s ability to compete with developed countries in the field of genetic technology research and development (discussed or mentioned in nine articles) (see Table 2). In contrast, only 9.8% of the articles (discussed or mentioned in four articles) argued against the implementation of human gene patents in China. The top cited reason is that gene patents may have adverse effects on China’s public health (discussed or mentioned in two articles) (see Table 2).

**Table 2:** Reasons for and against gene patents in China

Reasons for gene patents in China	Mentioned times
• Promoting inventions	18
• Protecting inventors	14
• Protecting genetic resources	12
• Competing with developed countries	9
• Fighting for genetic resources	5
• Maintaining Chinese bioengineering industry in the twenty-first century	3
• Helping translate technologies to business/profits	3
Reasons against gene patent in China	
• Protection of public health	2
• Genes are owned by human beings	1
• Chinese genetic resources have been drained by developed countries and have been patented in other countries. If China permits gene patents, Chinese will have to pay heavily to buy the patented Chinese genes back.	1

### Suggestions for the improvement of Chinese gene patent regime

Finally, 50.6% of articles (*n* = 40) provide recommendations for the improvement of the protection of intellectual property rights for gene patents in China. The proposed legal and policy recommendations can be summarized into four categories: 1) improvements in the standards for granting a gene patent; 2) increased concerns for the protection of human rights in the gene patent system; 3) protection of the interests of providers of genetic resources, and 4) establishment of a patent pool. The most commonly proposed suggestion was to increase the importance of the standard for practical applicability in the assessment of gene patent applications, which was suggested in 12 articles. In addition, nine articles argued that the gene patent regime should protect the interests of the providers of human genetic resources, insisting that providers should be able to use the patented genes for free. One article suggested that a mandatory licensing mechanism be implemented in situations that involve public health and policy.

## Discussion

The finding of this study indicates that debates about human gene patents have captured the attention of Chinese scholars, and that discussions have explored important issues associated with the patenting of human genes, e.g., benefits and potential risks, patent-eligibility of human genes, as well as challenges for the Chinese patent regime and the proposed improvements. It is noteworthy that the gene patent controversy, though, primarily a matter of legal issues, has drawn considerable contributions from disciplines other than the legal profession such as liberal arts, health sciences, and, in particular, related government departments, i.e., intellectual property offices at different levels. The wide-ranging participation in the debate indicates the complex nature of the issue. At the same time, it also demonstrates that patent protection for genes involves a variety of stakeholders in the field of genetic technology innovation and its commercialization.

Chinese scholars have contributed relatively balanced analyses of both benefits and risks associated with gene patents. However, they have been overwhelmingly in favor of the patenting of human genes in China. Some of the cited benefits and reasons for supporting a Chinese patent regime for genes are commonly mentioned in the western literature when discussing patent protection for human genes, e.g., promoting inventions and protecting inventors [23]. Nonetheless, some of the arguments made by Chinese scholars are unique and consider the relationship between developing countries, such as China, and developed countries. For example, when discussing the potential benefits, many Chinese scholars claimed that gene patents can help China to take a leader role in gene-related research and industry, or gene patents can increase China’s ability to compete with developed countries. The spirit of competition may reflect Chinese scholars’ mission in the field of biotechnology research and development and their understandings of the patent protection’s critical role in promoting the long-term development of China’s biotechnology field.

Except for the view that the patent regime can increase China’s competitiveness, Chinese scholars’ perspectives on the functions of a patent regime can also be perceived from other arguments they made. For example, a frequently raised rationale is that a patent regime can help to protect the genetic resources of developing countries. In particular, genetic data has been considered a critical part of national resources, and the phrase “genetic enclosure movement” appeared frequently in the articles, especially in those published in the early 2000s. These articles warned of the possibility that developed countries would use up Chinese genetic resources if a functional patent mechanism is not put in place. According to Chinese scholars, developing countries, in general, are rich in genetic resources but weak in technological research and development, while developed countries possess advanced technologies but with limited genetic resources. As a result, if developing countries do not have patent protection for human genes, developed countries can deprive China of its genetic resources easily and freely. This finding is consistent with research by Chen et al., that discusses the privacy protection of Chinese biobanks [24]. Their study indicates that a few incidents of bio-piracy that occurred in the late 1990s had alerted the Chinese government and heavily impacted later regulatory measures regarding genetic research and development in China [24]. In this regard, many Chinese scholars hope that using patent protection will prevent developed countries from free access to the Chinese genetic resources.

It is interesting that there is no change in the trend of Chinese scholars’ attitudes towards gene patents. They maintained a supportive position even after the US court’s landmark ruling in the 2013 *Myriad* case that isolated genes are not patent eligible. However, my research highlighted some nuanced perspectives on changes in Chinese scholars’ opinions about the scope of gene patents. For instance, before the 2010 *Guidelines*, when the patentability for gene patents was not clear, articles commonly encouraged recognitions of patentability of human genes by emphasizing the standard of practical application, one of the requirements when considering an approval for a gene patent under the *Patent Law of the PRC*. After 2010, scholars began to argue for a more rigorous examination of the practical applicability of the genes so as to limit the scope of gene patents. Such claims have been increasingly prominent in the article published after 2014. Although limiting the scope of the gene patent does not mean the denial of the patentability of human genes, the argument does indicate scholars’ considerations of the negative impact of a broad claim of a gene patent.

## Conclusions

This study explored the Chinese academic articles coverage of human gene patents. It aimed to examine not only scholars’ attitudes towards gene patents but also investigate their nuanced perspectives on the discussions of human gene patenting. As such, this paper explores the background of the author(s), benefits and risks associated with gene patents, reasons for and against the gene patent regime and the patentability of human genes, as well as the suggestions for improving Chinese IP system for the patenting of genes. This paper also discusses the rationale for the Chinese academic articles’ overwhelmingly support for human gene patents in China.

This study indicates that the development of genetic technology has been considered an important component of Chinese national economic development. Chinese academic scholars have engaged in the debate over human gene patents, and the majority of their published academic articles are supportive of the patenting of human genes in China. In general, Chinese scholars view a patent regime as an important legal tool to protect the interests of inventors and inventions as well as the genetic resources of China. In this regard, the patent regime has been accepted as a pipeline to develop the national economy and occupy genetic resources. The instrumentalism of the patent regime is so favored that even the risks and benefits of gene patents have been discussed by Chinese scholars in a balanced manner, yet they still prefer to support a gene patent system in China. This content analysis did not identify any changes in Chinese scholars’ attitudes toward gene patents after the court’s ruling in the *Myriad* case in 2013, but it is noteworthy that an increase in the value of practical application, the standard for granting a gene patent protection, has been suggested so that the scope of gene patents can be narrowed and their negative impact on public health can be mitigated.

## Abbreviations

BGI: Beijing Gene Institute
BRCA 1: Breast cancer genes 1
DNA: deoxyribonucleic acid
PRC: People’s Republic of China
